# Supplementary material for: Microwave-Assisted Synthesis of SPION-Reduced Graphene Oxide Hybrids for Magnetic Resonance Imaging (MRI)
Source: Nanomaterials (Basel). 2019 Sep 24;9(10):1364. doi: 10.3390/nano9101364 (PMC6835838; doi:10.3390/nano9101364)
Supplement: Supplementary file 1 [file nanomaterials-09-01364-s001.pdf]

nanomaterials

Supplementary

Microwave-Assisted Synthesis of SPION-Reduced Graphene Oxide Hybrids for Magnetic Resonance Imaging (MRI)

Marina Llenas [1], Stefania Sandoval [1,*], Pedro M. Costa [2], Judith Oró-Solé [1], Silvia Lope-Piedrafita [3,4], Belén Ballesteros [5], Khuloud T. Al-Jamal [2,*] and Gerard Tobias [1,*]

1 Institut de Ciència de Materials de Barcelona (ICMAB-CSIC), Campus de la UAB, 08193 Bellaterra (Barcelona), Spain; mllenas@icmab.es (M.L.); oro@icmab.es (J.O.-S.)
2 Institute of Pharmaceutical Science, King's College London, London, SE1 9NH, UK; pedrocosta24@gmail.com (P.M.C.)
3 Servei de Ressonància Magnètica Nuclear, Universitat Autònoma de Barcelona, Campus UAB, 08193 Bellaterra (Barcelona), Spain; silvia.lope@uab.es (S.L.-P.)
4 Centro de Investigación Biomédica en Red-Bioingeniería, Biomateriales y Nanomedicina (CIBER-BBN), Universitat Autònoma de Barcelona, Campus UAB, 08193 Bellaterra (Barcelona), Spain
5 Catalan Institute of Nanoscience and Nanotechnology (ICN2), CSIC and the Barcelona Institute of Science and Technology, Campus UAB, 08193 Bellaterra (Barcelona), Spain; belen.ballesteros@icn2.cat (B.B.)

* Correspondence: ssandoval@icmab.es (S.S.); khuloud.al-jamal@kcl.ac.uk (K.T.A.-J.); gerard.tobias@icmab.es (G.T.)

Received: 06 August 2019; Accepted: 18 September 2019; Published: 24 September 2019

1. Statistical analysis of NPs size (SPION) and RGO sheets area

Table S1. Descriptive analysis of diameter distribution of SPION and area distribution of RGO sheets.

	Num. of measures	Median	Lower adjacent observation	Q1 25th percentile	Q3 75th percentile	Upper adjacent observation	Max. observation	Average
**SPION**	596	7.14 nm	2.35 nm	5.59 nm	9.23 nm	14.56 nm	29.07 nm	8.03 nm
**RGO**	145	9.16 μm^2	0.23 μm^2	2.54 μm^2	25.97 μm^2	60.91 μm^2	78.26 μm^2	17.37 μm^2

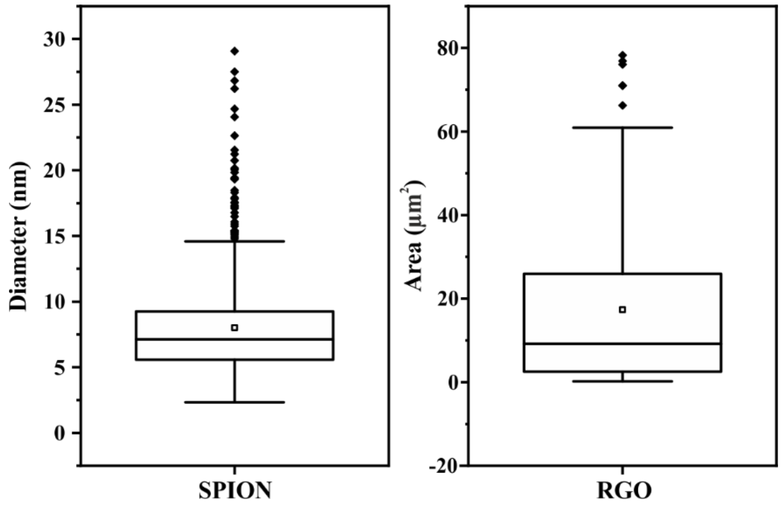

Figure S1. Box plot analysis of the diameter distribution of SPION and area distribution of RGO. Black rhombus indicates the outliers. The average value in each case is presented as the empty box in the graph.

2. Scanning electron microscopy images

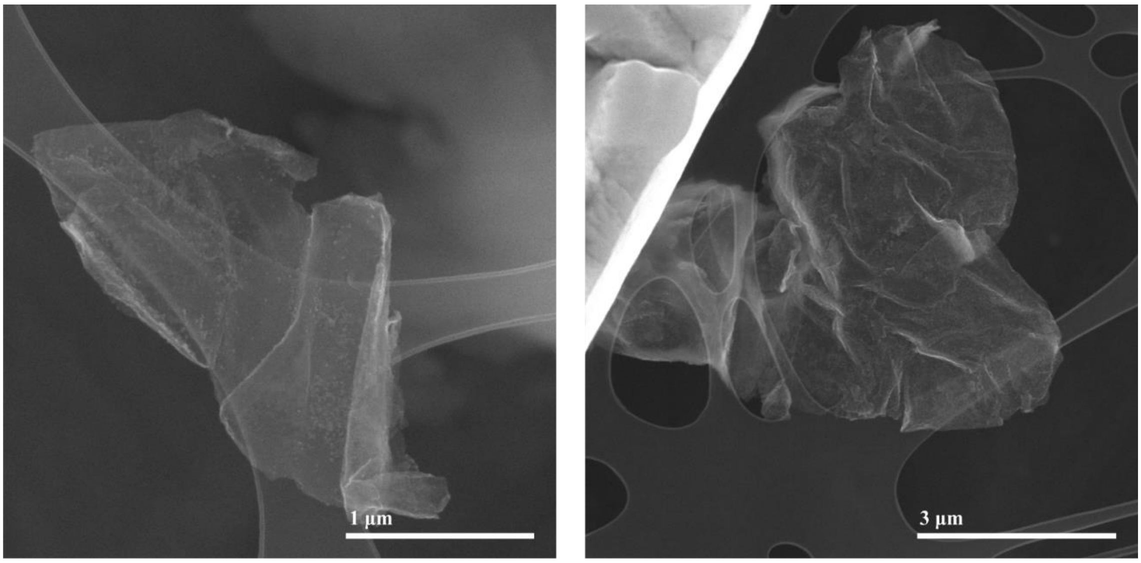

Figure S2. Scanning electron microscopy images of the SPION-RGO composites.

3. EFTEM compositional maps

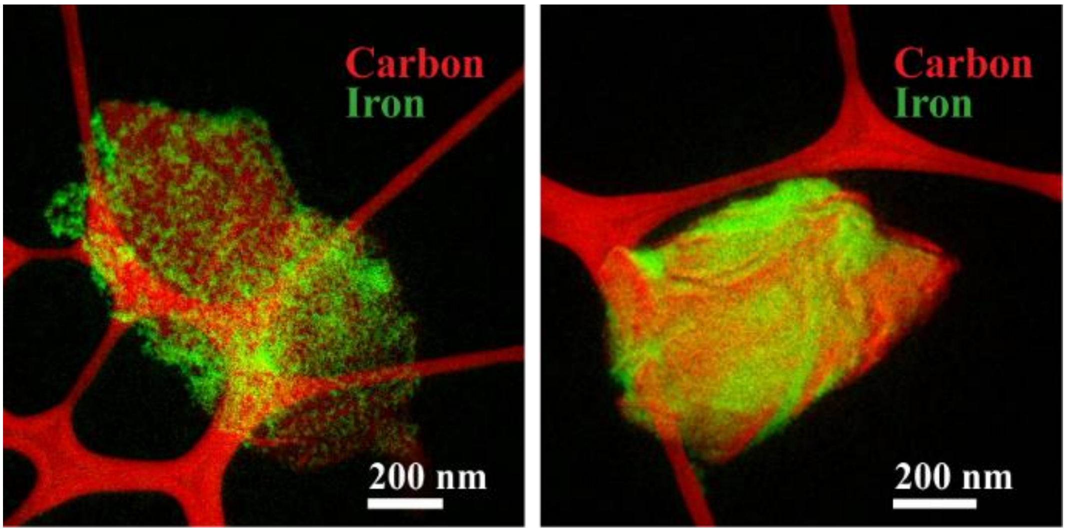

Figure S3. EFTEM compositional maps of the hybrid, where C and Fe appear in red and green respectively.

4. Transmission electron microscopy images

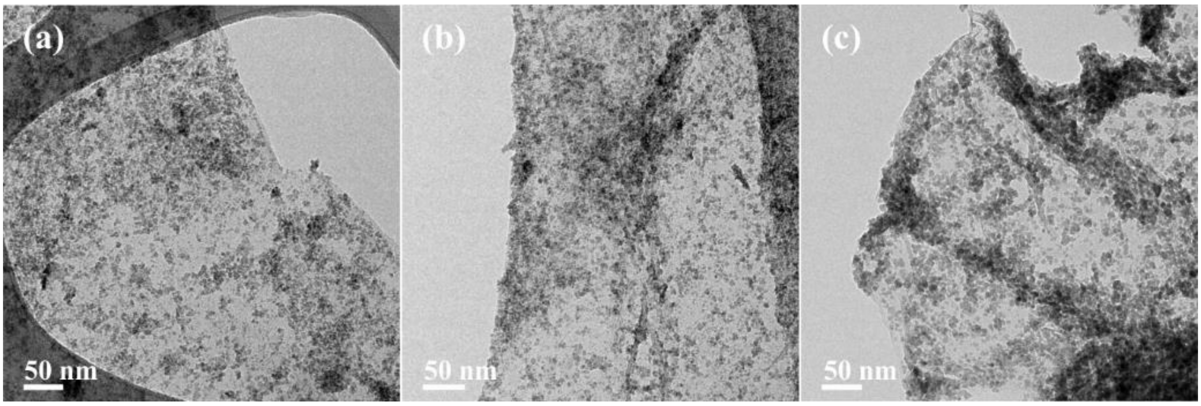

Figure S4. TEM images of SPION-RGO samples prepared by microwave-assisted reaction of 15 mg of graphene oxide and (a) 25 mg, (b) 30 mg and (c) 35 mg of iron(III) acetylacetonate.

5. Thermogravimetric analyses

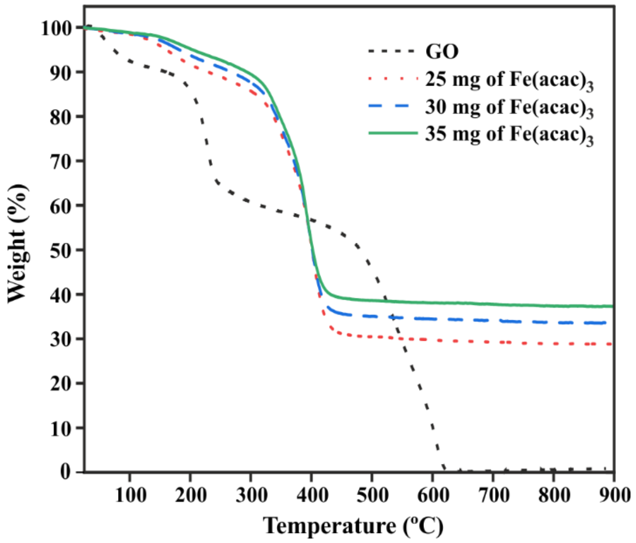

Figure S5. Thermogravimetric analyses of GO and SPION-RGO samples prepared using different amounts of iron precursor. TGA was performed under flowing air at a heating rate of 10 °C·min^{-1}.

6. Magnetic properties (300 K)

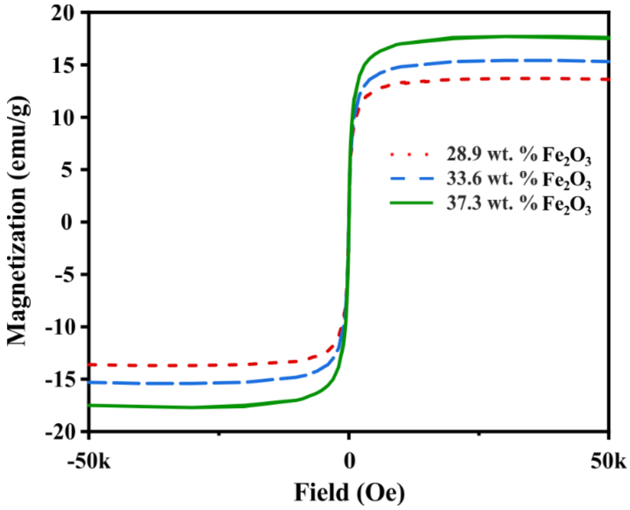

Figure S6. SPION-RGO hysteresis loops (magnetization versus field) at 300 K.

7. Phantom MRI Studies

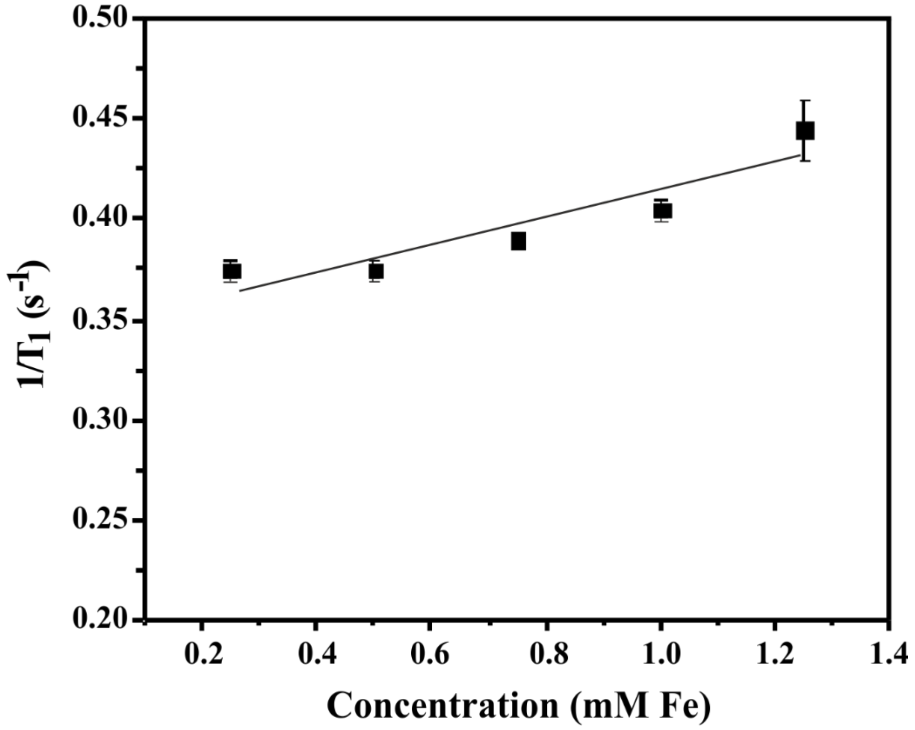

Figure S7. Phantom MRI studies of SPION-RGO composites, R_1 relaxation rates ($1/T_1$) versus Fe concentration. The relaxivity value was obtained from the slope, r_1 = 0.068 $mM^{-1}\cdot s^{-1}$. Results are the mean value ± S.D. (n = 2).
